# Self-supported β-Ga_2_O_3_ nanowires and for stretchable solar-blind UV photodetectors

**DOI:** 10.1038/s41598-025-02563-1

**Published:** 2025-05-20

**Authors:** Wuxu Zhang, Ziyin Xiang, Tengfei Ma, Baoru Bian, Jinyun Liu, Yuanzhao Wu, Yiwei Liu, Jie Shang, Run-Wei Li

**Affiliations:** 1 Ningbo Institute of Materials Technology and Engineering, Chinese Academy of Sciences, Ningbo, 315201 China; 2 College of Information and Intelligence Engineering, Zhejiang Wanli University, Ningbo, 315100 China; 3 Eastern Institute of Technology, Ningbo, 315200 China; 4 College of Materials Science and Opto-Electronic Technology, University of Chinese Academy of Sciences, 100049 Ningbo, China

**Keywords:** Self-supported, β-Ga_2_O_3_ nanowires, Stretchable, Solar-blind UV photodetectors, Nanowires, Optical sensors, Sensors and biosensors, Nanowires

## Abstract

Fire rescue robots equipped with solar-blind UV photodetectors can significantly improve operational safety and efficiency in hazardous environments involving high temperatures, explosions, toxic gases, and smoke. However, conventional rigid photodetectors cannot meet the flexibility requirements for seamless integration with robotic systems. This study presents a breakthrough in developing stretchable solar-blind UV photodetectors through an innovative combination of electrospinning and high-temperature phase transformation. Our approach enables high-yield production of self-supporting β-Ga₂O₃ nanowires with excellent solar-blind UV absorption. The fabricated metal-semiconductor-metal (MSM) photodetector demonstrates outstanding performance, including a remarkable photo-to-dark current ratio of 147.2 and responsivity of 22.5 mA/W. More importantly, the device exhibits unprecedented mechanical stability, with merely 3.4% photocurrent variation under 50% tensile strain and only 3.9% degradation after 500 strain cycles. We further demonstrate the practical applicability of this technology by fabricating a functional photodetector array capable of solar-blind UV imaging, paving the way for next-generation intelligent firefighting systems.

**Supplementary Information:**

The online version contains supplementary material available at 10.1038/s41598-025-02563-1.

## Introduction

 The fire rescue robot integrates modern intelligent and automated technologies, designed to replace traditional firefighters in entering fire scenes for firefighting and rescue operations^1,2^. These robots can operate in high-risk environments, such as those with high temperatures, explosive materials, toxic substances, or lack of oxygen, replacing firefighters and thus preventing injuries or fatalities. They play an increasingly important role in fire rescue operations, not only improving rescue efficiency but also ensuring the safety of firefighters. Flame detection is one of the core technologies that enables fire rescue robots to achieve intelligence and automation. It is primarily implemented through photoelectric detectors, image recognition sensors and temperature sensors^3–5^. Compared with image recognition sensors and temperature sensors, photodetectors have the advantages of fast recognition speed, high recognition accuracy and strong anti-interference ability, which is a hot spot for research in this field^6^. However, although the spectral range of the flame is relatively wide, covering the entire band of ultraviolet (105–380 nm), visible (380–700 nm) and infrared (700–1000 nm), due to the fact that only 200–280 nm solar blind UV light in daylight is absorbed by the atmospheric ozone layer, only solar blind UV photodetectors based on this band are less affected by natural light, they obtain signals with high signal-to-noise ratios and low false alarm rates, and have been the dominant means of flame detection^7–9^. At present, most of the solar-blind UV photodetectors are prepared by AlGaN^10^, diamond^11^ and Ga_2_O_3_^12^, but due to the difficulty in the preparation of AlGaN and the narrow response band of diamond, Ga_2_O_3_, which is thermally stable and has a high degree of solar-blind UV absorption, is the preferred material for the current solar-blind UV photodetectors^13^, which are mainly presented in the form of blocks, thin films, and nano-materials^12–14^, and Ga_2_O_3_ nanowires are highly valued because of their high specific surface area and good transport properties, which have attracted much attention^14^. In addition, Ga_2_O_3_ has five isomers, α, β, γ, ε and δ phases, of which the β phase is the most stable at room temperature and pressure^15–17^, all other phases can be transformed to the β-phase under certain conditions, making β-Ga_2_O_3_ nanowires an ideal material for realizing solar-blind UV light detection. In order to enable the solar blind UV photodetector to be integrated with the fire rescue robots with complex curved surfaces in a conformal manner, and to meet the deformation capability requirements of the robots when working and the accuracy of detection during deformation, there is an urgent need to develop a flexible solar blind UV photodetector. For this reason, in recent years, researchers have prepared bendable solar-blind UV photodetector by using amorphous or β-phase Ga_2_O_3_ nanowires or thin films as functional layers on bendable and flexible substrates such as mica^18^, PET^19^, PEN^20^, and fiberglass fabrics^21^, etc. However, due to the weak deformation ability and the poor strain stability, they are still not able to satisfy the application requirements of fire rescue robots.

The development of stretchable solar-blind UV photodetectors requires effective integration of β-Ga₂O₃ nanowires with elastic substrates. While chemical vapor deposition (CVD) can produce β-Ga₂O₃ nanowires, its high-temperature requirements (≥ 800 °C)^15,22,23^ are incompatible with temperature-sensitive elastic substrates (tolerance < 200 °C)^24,25^. Furthermore, the vapor-liquid-solid (VLS) growth mechanism^26^ yields limited quantities of nanowires, making it unsuitable for large-scale production of self-supported films. To overcome these limitations, we developed an innovative approach combining electrospinning with high-temperature phase transition, enabling mass production of high-quality β-Ga₂O₃ nanowires. The resulting metal-semiconductor-metal (MSM) photodetectors demonstrate exceptional performance, including a high photo-to-dark current ratio (147.2), responsivity (22.5 mA/W), and remarkable strain stability (merely 3.4% photocurrent variation at 50% strain). After 500 strain cycles, the devices maintain 96.1% of their initial response, making them ideal for integration into fire rescue robots and alarm systems where mechanical flexibility is crucial.

## Experimental section

### Material and instruments

The specifications and manufacturers of the materials and instruments used in this work are shown in Table 1.

**Table 1:** Experimental materials and instruments.

Name	Specification	Manufacturer
Gallium nitrate hydrate Ga(NO_3_)_3_·xH_2_O	99.99%	Macklin
Polyvinylpyrrolidone (PVP)	K-30	Macklin
Anhydrous ethanol	Analytical grade	Sinopharm Group, CN
Disposable syringe	5 mL/1.3 cm	Cofoe Medical
Stainless steel capillary tube	20 G/0.61 mm	Dexing Hardware, CN
Magnetic stirrer	/	Hefei Kejing, CN
Electrospinning machine	/	Beijing Yongkang, CN
Single-zone vacuum tube furnace	OTF-1200X	Hefei Kejing, CN

### Preparation of β-Ga₂O₃ nanowires

First, Ga(NO₃)₃·xH₂O and K-30 PVP, with a mass ratio of 1:4, are dissolved in an ethanol and deionized water solution (1 g Ga(NO₃)₃·xH₂O in 11 mL ethanol and 9 mL deionized water) to prepare a precursor solution. This solution is then mixed uniformly using a magnetic stirrer.

Next, the precursor solution is placed in a 5 mL disposable syringe with a diameter of 1.3 cm, and the needle is a stainless steel capillary tube with an inner diameter of 0.61 mm. Electrospinning is then performed to obtain precursor nanowires. The electrospinning equipment parameters are set as follows: electrode voltage at + 18 kV/−2 kV, solution feed rate at 0.05 mm/min, distance between the needle and the collector at 14 cm, and ambient temperature at 30 °C.

Finally, the self-supporting precursor nanowires are peeled off from the substrate and placed in a vacuum oven at 80 °C for 12 h to remove moisture. This step prevents the formation of hydroxide gallium (GaO(OH)) during high-temperature calcination by avoiding a humid environment. After drying, the precursor nanowires are subjected to vacuum calcination in a high-temperature tube furnace. The temperature is rapidly increased to 500 °C at a rate of 10 °C/min to prevent the formation of hydroxide gallium (GaO(OH)), and high-purity oxygen is continuously supplied. The temperature is then slowly raised to 900 °C at a rate of 5 °C/min, and once the target temperature is reached, high-purity oxygen is supplied continuously for 2 h of calcination. This calcination process is repeated multiple times to obtain β-Ga₂O₃ nanowires with high UV absorption in the blind region, which are then used to fabricate stretchable UV photodetectors. For detailed steps in the preparation of β-Ga₂O₃ nanowire materials, refer to Supplementary Material Figure S1.

### Preparation of stretchable solar-blind UV photodetector with β-Ga_2_O_3_ nanowires

Considering the Young’s modulus and solar-blind UV transmittance of elastomers, PDMS was selected as the elastic substrate for the stretchable solar-blind UV detector, and the comparison of different elastic substrates is shown in Supplementary Material Figure S2. The Ag nanowire stretchable electrode was prepared by filtration, which has good contact stability with β-Ga_2_O_3_ nanowires in staggered overlap. In order to make the filtration more efficient and prevent the Ag and β-Ga_2_O_3_ nanowires from passing through during the filtration, the filter paper was made of PC polycarbonate filter membrane with a pore size of 0.22 μm (Millipore’s GTTP04700). First, the filter membrane was laid flat on the filter extractor, and the negative pressure was pumped inside the filter extractor bottle, the pre-prepared PDMS-based mask template with a thickness of 50 μm was covered on the filter membrane, and the Ag nanowire solution was added drop-by-drop to the vacancies on the mask template, and then the drop addition was stopped after the formation of a thin layer, and the PDMS mask template was taken off after the solution was completely dried up. Next, the β-Ga₂O₃ nanowires obtained from the high-temperature phase transition are dissolved in a small amount of ethanol and then drop-cast between two electrodes. Once a thin film is formed, the drop-casting is stopped, resulting in a film with an overall thickness of approximately 50 μm. Finally, the device is encapsulated with uncured PDMS in a 1:10 mass ratio, and placed in a forced air oven at 60 °C for curing for at least 3 h. After curing, the filter membrane is removed, and the PDMS elastic substrate is cut into a 10 mm × 50 mm rectangle, resulting in a stretchable device.

### Characterization of the performance of stretchable solar-blind UV photodetector with β-Ga_2_O_3_ nanowire

The tensile strain was applied by a precision handwheel T-slide of the strain application system, as shown in Figure S3, and the optoelectronic performance test was based on a self-constructed optoelectronic test system based on B1500 A and a 254 nm light source, as shown in Figure S4 of the supplementary material.

The performance of stretchable solar-blind UV photodetectors is usually evaluated using indicators such as photocurrent, dark current, photo-to-dark current ratio, response time and recovery time, and responsivity, which are described separately below:

1. Photocurrent ($\:{\text{I}}_{\lambda}$) and dark current ($\:{\text{I}}_{\text{dark}}$)

The physical meaning of photocurrent is the current measured by the device under solar-blind UV irradiation with or without a certain bias voltage. The physical meaning of dark current is the current measured by the device in a completely dark dark box environment^15^. The unit is generally expressed in amperes (A), and the photocurrent of the device is related to the optical power density and the applied bias voltage.

(2) Photo-to-dark Current Ratio (*PDCR*).

PDCR is the ratio of photocurrent to dark current, which is calculated as shown in Eq. 1 since the current measured under light conditions is actually the sum of photocurrent and dark current.

 

1 $$\:PDCR=\left({\text{I}}_{\lambda}\:\text{-}\:{\text{I}}_{\text{dark}}\right)/{\text{I}}_{\text{dark}}$$

where $\:{\text{I}}_{\lambda}$ is the response current of $\:\lambda\:$ wavelength light and $\:{\text{I}}_{\text{dark}}$ is the dark current in a dark environment.

(3) Response time and recovery time.

Response time, also known as rise time, is the time taken for the current of the device to rise from 10 to 90% of the peak photocurrent, starting from the time of applying light at a fixed bias voltage. The recovery time, also known as the fall time, is the time taken for the current of the device to fall from 90 to 10% of the peak photocurrent at a fixed bias voltage, starting from the time when the light is withdrawn^20^. The response time of the device is usually affected by the defect state of the material, with more defective devices usually having longer response times.

(4) Responsivity (*R*).

*R* represents the relationship between the incident optical power of the photodetector and the output photocurrent, often expressed in amperes per watt (A/W)^27^^[,28^, with the expression shown in Eq. 2.

2 $$\:R=\left({\text{I}}_{\lambda}\:\text{-}\:{\text{I}}_{\text{dark}}\right)/{(P \times S)}$$

where $\:{\text{I}}_{\lambda}$ is the response photocurrent at $\:\lambda\:$ wavelength, $\:{\text{I}}_{\text{dark}}$ is the dark current in a dark environment, *P* is the incident light power, and *S* is the effective light-sensitive area of the device.

## Result and discussion

### Characterization of β-Ga_2_O_3_ nanowires

**Fig. 1:**
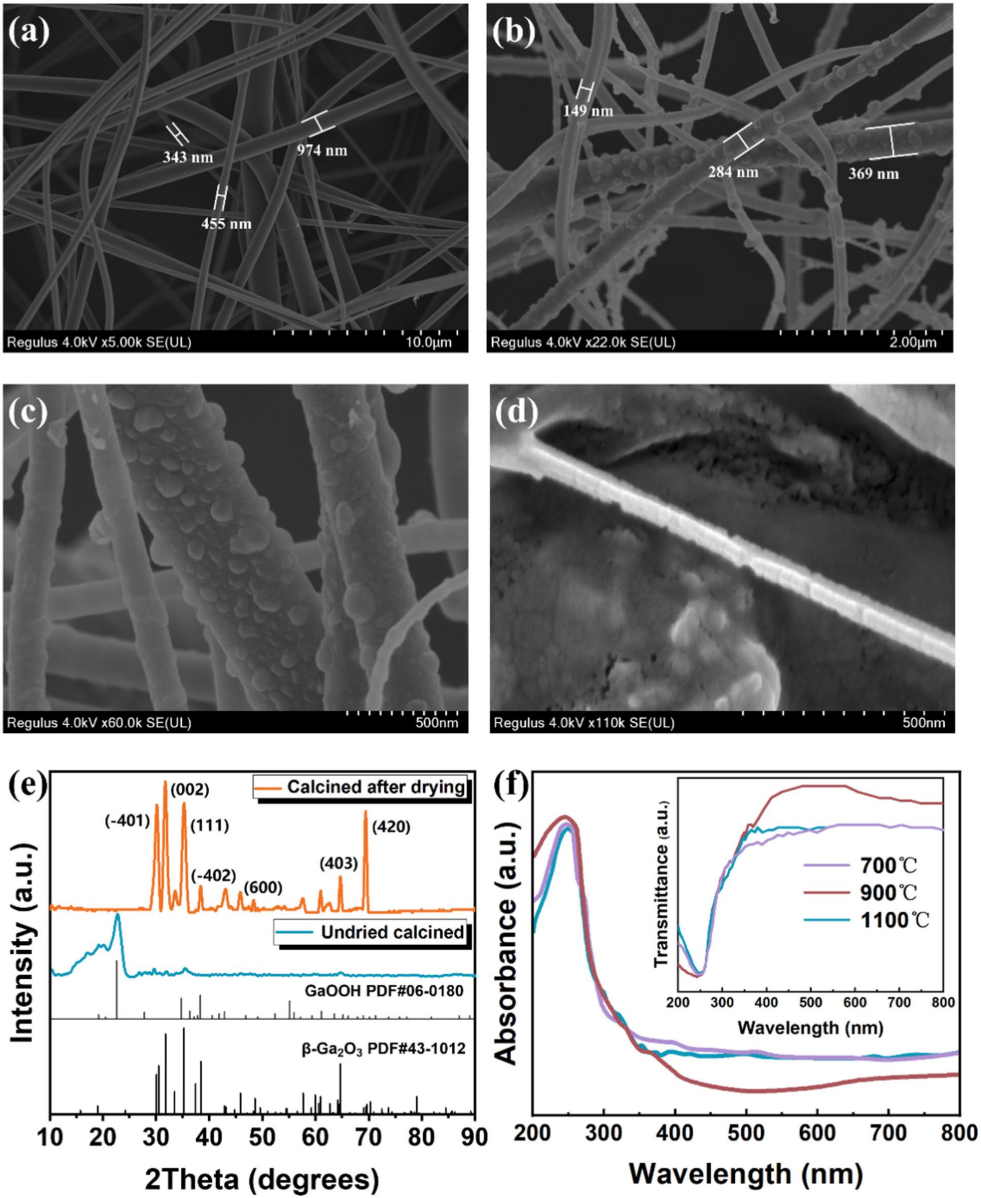
Characterization of β-Ga₂O₃ Nanowires. (**a**) Microscopic morphology of the precursor nanowires after electrospinning and drying. (**b**) Microscopic morphology of Ga₂O₃ nanowires obtained after high-temperature phase transition. (**c**) Surface of the nanowires showing noticeable dense protrusions after calcination. (**d**) Microscopic morphology of pure β-Ga₂O₃ nanowires after multiple calcinations and removal of C residues. (**e**) Comparison of XRD patterns of materials obtained from dried calcination versus direct calcination. (**f**) Comparison of transmission spectra of materials obtained at different calcination temperatures.

The nanowires prepared by the electrostatic spinning method were characterized and analyzed by SEM, XRD, EDS and UV-Vis. The morphology of precursor nanowires prepared by electrostatic spinning after vacuum oven drying is shown in Fig. 1(a), and the microscopic morphology of nanowires after high temperature calcination is shown in Fig. 1 (b). From Fig. 1 (c), it can be observed that after calcination, the surface of the nanowires shows a noticeable distribution of dense protrusions. EDS testing was conducted on individual protrusions of a single nanowire, as detailed in Supplementary Material Figure S5. It was found that in addition to Ga and O elements, C elements were also present. Given that polyvinylpyrrolidone (PVP) contains C, H, N, and O elements, it can be concluded that the C element is a residue from the high-temperature decomposition of PVP. The nanowires were subjected to multiple calcination processes. After each calcination, high-purity oxygen was continuously introduced once the temperature reached above 500 °C. When the temperature reached 900 °C, high-purity oxygen was continuously supplied for 2 h to perform the calcination. This high-temperature calcination in a high-purity oxygen environment was intended to remove the carbon residues produced from the high-temperature decomposition of PVP. From Fig. 1(d), it can be observed that after high-temperature oxidation, scattered pits appear on the surface of individual nanowires. Additionally, the disappearance of C elements in the selected-area EDS electron diffraction indicates that, compared to before multiple calcinations, the high-temperature oxidation has effectively removed the C residues from the individual nanowires. To verify that the precursor nanowires have transformed into β-Ga₂O₃ nanowires, X-ray diffraction (XRD) analysis was conducted on the material obtained after vacuum drying and high-temperature oxidation at 900 °C. The XRD pattern is shown in Fig. 1(e). The orange XRD pattern in Fig. 1(e) reveals seven strong diffraction peaks at 2θ values of 30.5°, 31.7°, 35.2°, 38.4°, 45.8°, 64.7°, and 69.4°. Comparing with the standard data card for β-Ga₂O₃ (JCPDS#43–1012), these peaks correspond to the crystal planes (−401), (002), (111), (−402), (600), (403), and (420), indicating that the material obtained after high-temperature oxidation of the precursor nanowires is monoclinic β-Ga₂O₃. However, the presence of numerous diffuse diffraction peaks suggests that the material is not purely monoclinic β-Ga₂O₃, and the bulging in the XRD pattern indicates possible presence of some amorphous phases. Compared to the XRD pattern of the material obtained by direct high-temperature calcination without vacuum drying (blue line in Fig. 1(e)), the diffraction peaks of GaO(OH) have significantly disappeared, and the intensity of the diffraction peaks corresponding to β-Ga₂O₃ nanowires has increased. This indicates that vacuum drying before high-temperature calcination effectively prevents the formation of GaO(OH), confirming the necessity of the vacuum drying step.

To study the effect of different calcination temperatures on the ultraviolet absorption and optical band gap of β-Ga₂O₃ nanowires, the precursor nanowires, after vacuum drying, were calcined at 700 °C, 900 °C, and 1100 °C, respectively, followed by high-temperature oxidation to remove carbon. The resulting materials were then tested using UV-Vis spectroscopy. The transmission and absorption spectra of the materials obtained at different calcination temperatures are shown in Fig. 1(f). The absorption spectra and transmission spectra of the β-Ga₂O₃ nanowires prepared at different temperatures indicate that all samples exhibit high absorption and low transmission in the ultraviolet range of 200–280 nm, with an absorption cutoff edge at around 270 nm. In the visible light range of 400–760 nm, the materials show almost no absorption and nearly complete transmission, demonstrating some degree of suppression of both ultraviolet and visible light. This property is advantageous for the fabrication of ultraviolet detectors. The optical band gaps of the β-Ga₂O₃ nanowires prepared at different calcination temperatures were estimated from the (αhv)² versus hv plots, as detailed in Supplementary Material Figure S6. The energy values corresponding to the intersection points of the extrapolated lines with the x-axis are the estimated optical band gaps of the samples, which are 4.41 eV (700 °C), 4.60 eV (900 °C), and 4.64 eV (1100 °C), all within the band gap range required for ultraviolet detection. The band gap of the material calcined at 700 °C is at the edge of the ultraviolet detection range, while the optical band gap of the material calcined at 1100 °C is similar to that at 900 °C. An increase in calcination temperature enhances the crystallinity of β-Ga₂O₃ nanowires while reducing the amorphous phase content. Amorphous Ga₂O₃ typically exhibits a lower bandgap (~ 4.0–4.5 eV) due to its disordered structure, which leads to electronic state tailing (Urbach tail effect). In samples synthesized at lower temperatures (700 °C), residual amorphous phases may contribute to a reduced overall bandgap. In contrast, high-temperature calcination (900–1100 °C) promotes the transformation of the amorphous phase into crystalline β-Ga₂O₃^29^, which possesses a higher intrinsic bandgap (~ 4.7–4.9 eV), thereby increasing the material’s effective bandgap. Furthermore, low-temperature processing may result in the formation of metastable phases (e.g., α-Ga₂O₃, with a bandgap of ~ 5.0 eV) or incompletely reacted GaO _x_ impurities^30^, whereas high-temperature treatment facilitates the formation of phase-pure β-Ga_2_O_3_ Additionally, elevated temperatures enhance oxygen diffusion, driving the material closer to its stoichiometric composition (Ga_2_O_3+x_ O_3,+x_ where x→0), which mitigates bandgap narrowing caused by oxygen deficiency. However, the material calcined at 900 °C has lower absorption in the visible light range of 400–760 nm, theoretically providing better suppression of ultraviolet and visible light. Therefore, 900 °C is considered the optimal calcination temperature.

### Photoelectric performance analysis of stretchable solar-blind UV photodetector with β-Ga_2_O_3_ nanowire

**Fig. 2:**
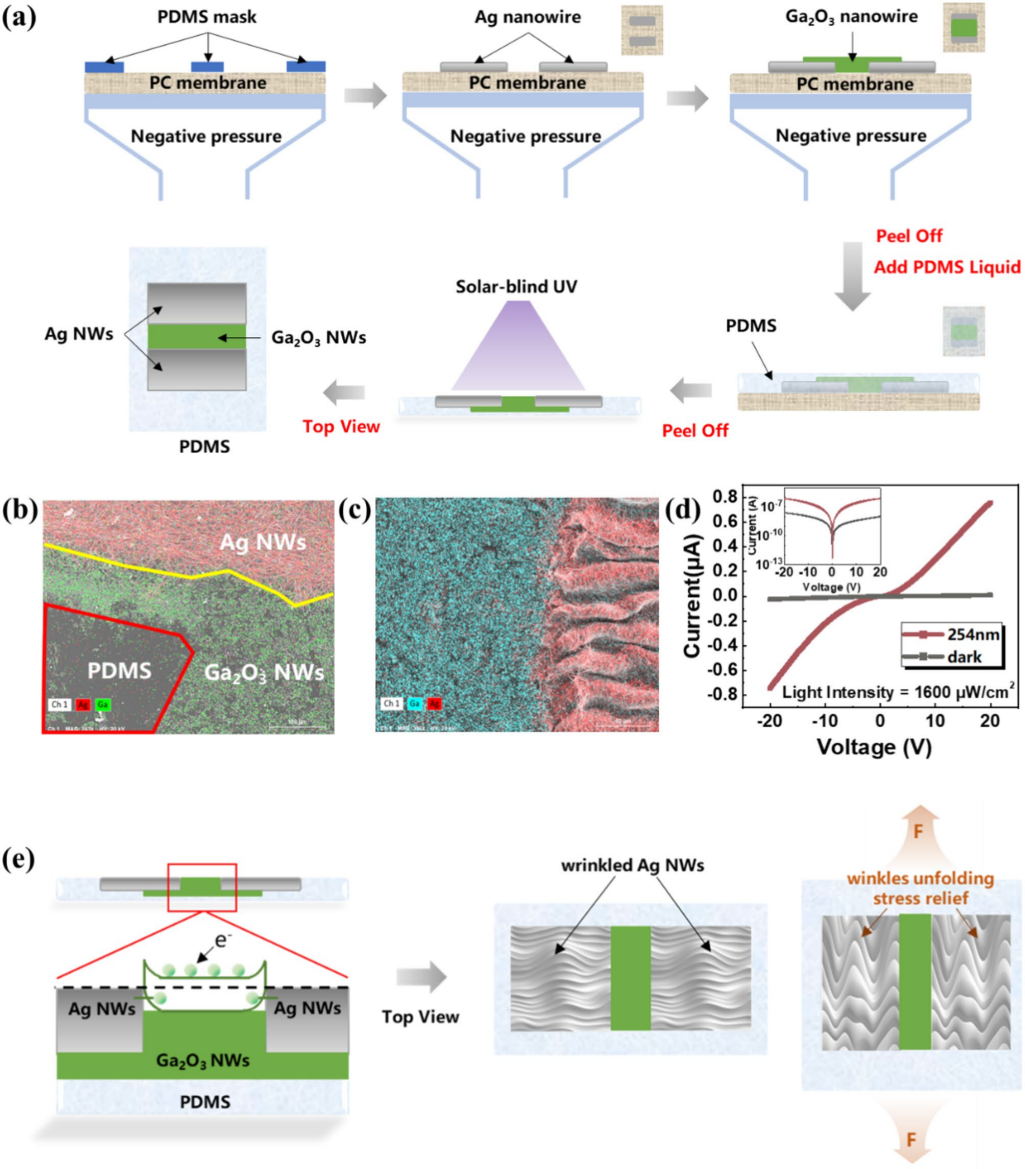
Fabrication and Characterization of Stretchable UV Photodetectors Based on β-Ga₂O₃ Nanowires. (**a**) Flowchart of the fabrication process for the stretchable UV photodetector based on β-Ga₂O₃ nanowires and Ag nanowires. (**b**) Surface scanning EDS of the contact boundaries between the Ag NWs electrodes, PDMS substrate, and Ga₂O₃ NWs. (**c**) Surface scanning EDS of the contact boundaries between the Ag NWs electrodes and β-Ga₂O₃ NWs. (**d**) I-V characteristic curves of β-Ga_2_O_3_ nanowire stretchable day-blind UV detectors in the dark and under 254 nm illumination. (**e**) Schematic diagram of MSM solar blind UV detector carrier transfer schematic and how the local strain dissipates through the wrinkles of Ag NMS.

The MSM-type stretchable UV photodetector consists of three main components: an elastomer, a wide-bandgap semiconductor material, and electrodes. The preparation process for the stretchable UV photodetector based on β-Ga₂O₃ nanowires is illustrated in Fig. 2(a). The distance between the two electrodes of a single device is 1 mm, with each Ag nanowire electrode measuring 1 mm × 4 mm. The electrodes are covered with β-Ga₂O₃ nanowires measuring 4 mm × 1 mm. The photosensitive area of the stretchable UV photodetector based on β-Ga₂O₃ nanowires is approximately 0.04 cm².

To characterize the interface contact between the Ag nanowire electrodes and β-Ga₂O₃ nanowires, scanning electron microscopy (SEM) and surface scanning EDS analyses were performed on the contact boundaries, as shown in Fig. 2(b) and (c). The contact boundaries between the Ag nanowire electrodes, the PDMS substrate, and the β-Ga₂O₃ nanowires are clearly defined. The Ag nanowires and β-Ga₂O₃ nanowires interlock and overlap, forming good contact. Additionally, a large number of β-Ga₂O₃ nanowires can be observed both in cross-section and on the surface of the device, ensuring effective light sensing and current transfer. In the fabrication of flexible photodetectors, precise control of wrinkle morphology in silver (Ag) nanoelectrodes enhances their tensile stability and performance. Wrinkles naturally form at the interface between rigid Ag nanowires and elastomeric substrates like PDMS due to elasticity differences, acting as strain buffers. By adjusting the position and speed of droplet application during Ag nanowire deposition, the direction and size of these wrinkles can be controlled. Aligned wrinkles effectively distribute strain along the direction of applied stress, while small, uniform wrinkles maintain mechanical integrity and electrical conductivity by preventing nanowire breakage or misalignment. This controlled wrinkle morphology allows the electrodes to retain stable conductivity and structural integrity under repeated tensile cycles, making them ideal for flexible device applications requiring durability and reliability under strain.

The I-V characteristic curve is an important reference for evaluating the performance of UV photodetectors. Under dark conditions or with illumination of 1600 µW/cm² at 254 nm UV light, the I-V characteristic curves of the device at biases ranging from − 20 V to 20 V are shown in Fig. 2(d). At a bias of 20 V, the dark current of the device is 5.12 × 10⁻⁹ A, and at −20 V, the dark current is 2.49 × 10⁻⁸ A. The extremely low dark current indicates that the device has very high resistance before light is applied. Figure 2(d) shows a noticeable asymmetry in the dark current. The significant difference in dark current between forward and reverse bias is due to the Schottky barrier created by the difference in work functions between the electrode and the semiconductor. When positive and negative biases are applied, electrons in trap states are first captured and then released, causing the current during capture to be lower than during release. According to this theory, the more oxygen vacancy defects in the material, the more trap states there are, resulting in more pronounced asymmetry in the dark current. Similarly, the Schottky barrier helps the device achieve lower dark current. When the device is illuminated with 254 nm UV light, the photocurrent is 7.61 × 10⁻⁷ A at 20 V bias and 7.44 × 10⁻⁷ A at −20 V bias. From this data, it can be concluded that the stretchable UV photodetector based on β-Ga₂O₃ nanowires has a strong photoelectric response to UV light in the 254 nm blind UV range. In addition, Fig. 2(e) Showing how the MSM solar blind UV detector carrier transfer and how the pre-designed wrinkles accommodate strain through reversible unfolding while maintaining electrical contact, the interlocking β-Ga₂O₃ nanowire network that preserves charge transport pathways under deformation.

**Fig. 3:**
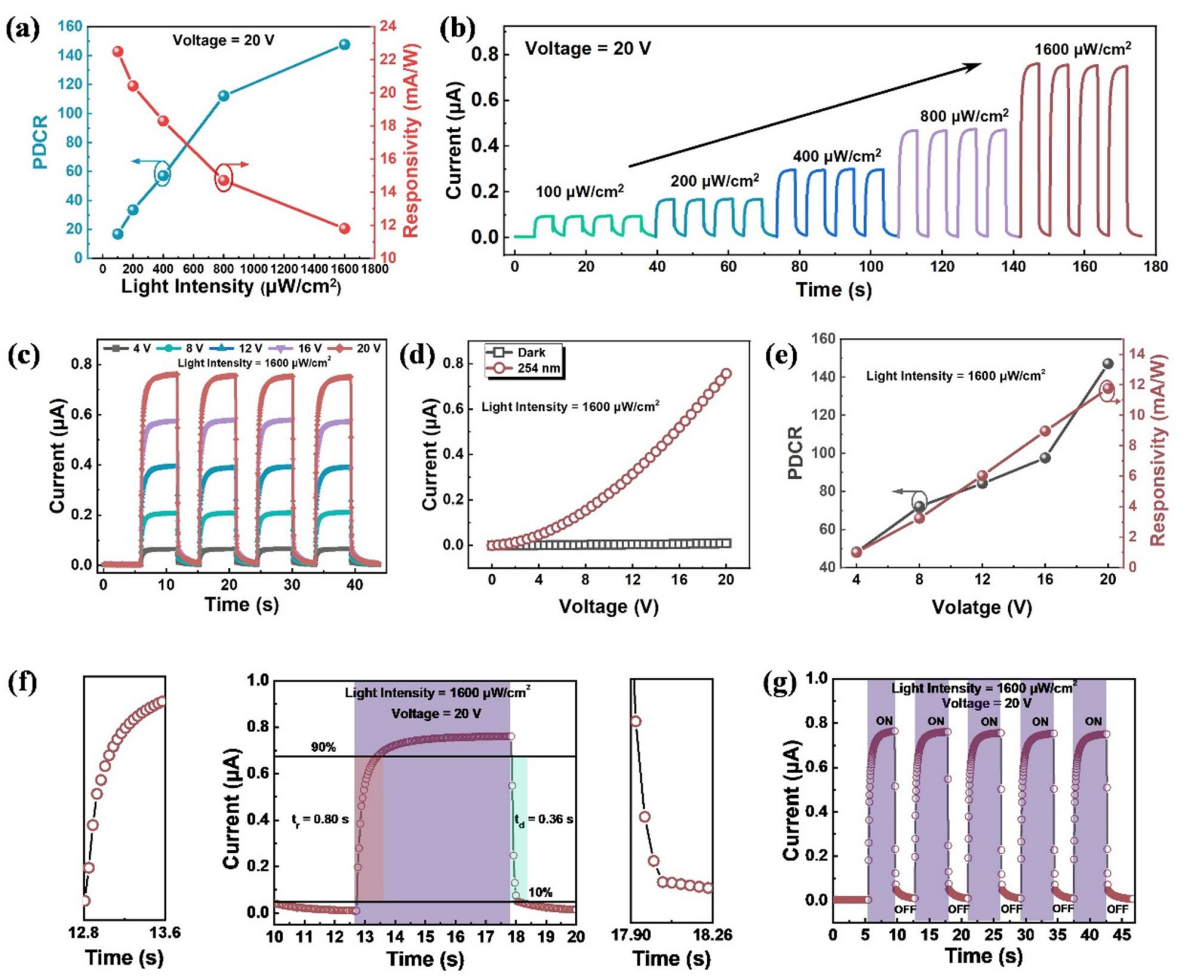
Photoelectric Performance of the Device in the Unstrained State. (**a**) Trends of responsivity (R) and *PDCR* with changes in light power density. (**b**) I-T cyclic response curves at different light power densities. (**c**) I-T characteristic curves at different bias voltages. (**d**) Relationship between photocurrent and applied bias. (**e**) Trends of responsivity (R) and *PDCR* with changes in bias voltage. (**f**) I-T curves and response time. (**g**) Multi-cycle I-T curves.

The *PDCR* and *R* are important indicators for evaluating the UV detection capability of the device. *PDCR* intuitively represents the change in current caused by illumination, while responsivity describes the relationship between the incident light power and the output photocurrent. The relationship between *PDCR* and light power density is shown in Fig. 3(a). It can be seen from the figure that when the incident light power density is 1600 µW/cm², the maximum *PDCR* of the device is 147.2. As the light power density increases, the *PDCR* of the device shows an upward trend. Increasing light power density generates more electron-hole pairs, leading to a larger photocurrent. However, as the light power density increases, the responsivity *R* decreases with increasing light power density, and the rate of decrease gradually slows down. When the incident light power density is 100 µW/cm², the device achieves a maximum responsivity of *R* = 22.5 mA/W. This is due to defects in the material generating trap states and electron-hole recombination centers, which cause the photocurrent of the device to increase sublinearly with light power density. Consequently, the increase in the numerator in Eq. 2 is slower than the increase in the denominator, which is the light power density (*P*), leading to a gradual decrease in responsivity with increasing incident light power.

Under a bias of 20 V, periodic illumination with 254 nm UV light at different light power densities (100–1600 µW/cm²) was applied, and the I-T characteristic curve of the device is shown in Fig. 3(b). At high light intensity (light power density of 1600 µW/cm²), the photocurrent of the device reaches 0.76 µA, while at low light intensity (light power density of 100 µW/cm²), the response current is still 0.095 µA, indicating that the device has a certain capability for detecting low light intensity UV. Within five cyclical periods at the same light power density, the peak photocurrent of the device remains stable and reproducible, showing differentiated response currents for UV light at different power densities. The I-T characteristic curve of the device provides information on the response time, and the cyclical testing reflects the stability of the device’s photoelectric response.

Applying a higher bias voltage facilitates the separation of electron-hole pairs generated by incident light, leading to an increased photocurrent and affecting the device’s photoelectric performance. The impact of different applied biases (4 V, 8 V, 12 V, 16 V, and 20 V) on the I-T characteristic curves of the device is shown in Fig. 3(c). Under periodic illumination with 1600 µW/cm² UV light, as the applied bias increases, the peak response current of the device gradually rises, while the peak response current remains stable at the same bias. The relationship between the peak photocurrent, dark current, and applied bias is shown in Fig. 3(d). As the bias increases, the device’s photocurrent response to 254 nm UV light also increases. At a light power density of 1600 µW/cm², the photocurrent is only 65 nA at 4 V bias, but reaches 0.758 µA at 20 V bias. This is because a higher bias makes it easier for the electron-hole pairs excited by UV light to separate, resulting in increased photocurrent, while the dark current remains almost unchanged with increasing bias. Consequently, the device’s *R* and *PDCR* as functions of bias voltage are shown in Fig. 3(e). As the bias increases, both *R* and *PDCR* increase to varying degrees. At a light power density of 1600 µW/cm², the responsivity is only 0.99 mA/W and the *PDCR* is 48.2 at 4 V bias, whereas at 20 V bias, the *R* reaches 11.8 mA/W and the *PDCR* reaches 147.2. This is mainly due to the higher bias facilitating the separation of more electron-hole pairs generated by light, thus producing a higher response photocurrent. The I-T characteristic curve was tested with a 5-second on-off cycle of 1600 µW/cm² UV light, controlled by a millisecond timer, at a 20 V bias, as shown in Fig. 3(f). The response time of the device is approximately 0.80 s, and the recovery time is approximately 0.36 s. Figure 3(g) shows the I-T curves for three cycles. It can be seen that when UV light is applied, the response current reaches a stable peak, and after removing the UV light, the current can return to the same order of magnitude as the initial dark current without affecting the I-T response curve of the next cycle. Additionally, the response time and recovery time for each cycle remain nearly constant, and the peak response current is stable, indicating that the device’s response is stable and reproducible.

The intensity-dependent responsivity (Fig. 3a, e) originates from the competition between carrier generation and defect-mediated recombination. At low light intensity (100 µW/cm²), the near-linear photocurrent and peak responsivity (22.5 mA/W) indicate dominant carrier separation with negligible recombination. The sublinear photocurrent growth and decreased R at high intensity (1600 µW/cm²) suggest trap-state saturation (e.g., oxygen vacancies) via Shockley-Read-Hall recombination. The Schottky barriers at Ag/β-Ga₂O₃ interfaces (Fig. 2d) further modulate carrier collection, while higher bias (20 V) suppresses recombination, enhancing R and PDCR.

The photoelectric performance of the stretchable solar-blind UV photodetector based on β-Ga₂O₃ nanowires in this work is compared with the solar-blind UV detectors based on β-Ga₂O₃ nanowires reported in the literature, as shown in Table 2. The main feature of this work is achieving stretchability without sacrificing various photoelectric performances. The following section will focus on the strain stability of the stretchable solar-blind UV detector based on β-Ga₂O₃ nanowires developed in this study.

**Table 2:** Comparison of the photoelectric performance of Solar-Blind UV photodetectors based on β-Ga₂O₃ nanowires.

	Photocurrent (µA)	PDCR	*R* [mA/mW]	Stretchability
^15^β-Ga₂O₃ nanorods-glass fiber fabric	0.0135	24.79	740	No
^22^ β-Ga₂O₃ nanowires-quartz substrate	2	10^3^	53.4	No
^31^β-Ga₂O₃/GaN-sapphire substrate	4	1.375 × 10^3^	27.5	No
^32^ β-Ga₂O₃ nanowires-Ga thin film	0.027	10^3^	2.9	No
^33^ β-Ga₂O₃ nanowires-sapphire substrate	22.5	10^4^	122	No
^34^ β-Ga₂O₃ nanoflakes- Si (001) substrate	912	2.1 × 10^4^	1403	No
[This work] β-Ga_2_O_3_-PDMS	0.76	147.2	22.5	Yes

### Strain stability analysis of stretchable solar-blind UV photodetectors with β-Ga₂O₃ nanowires

**Fig. 4:**
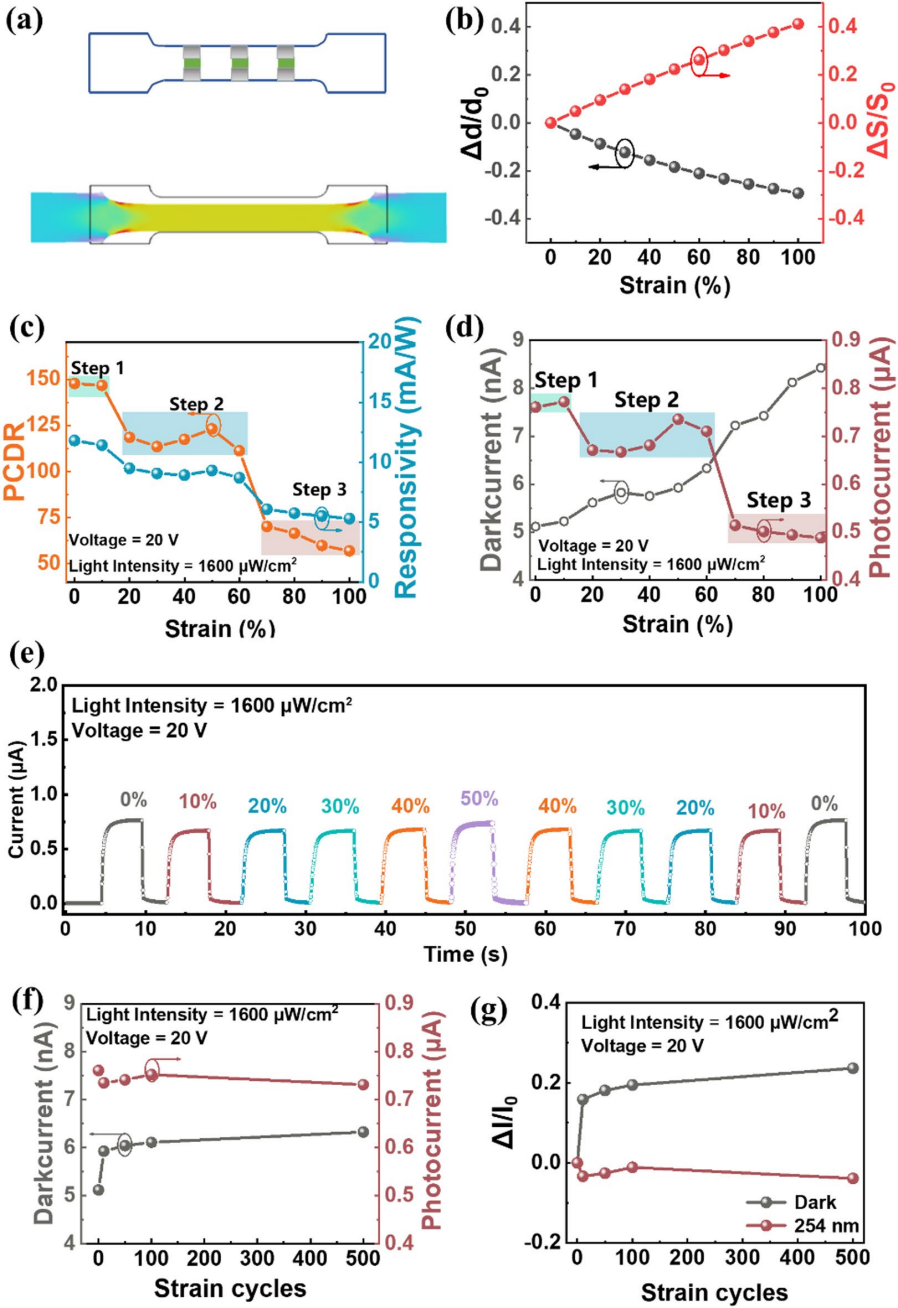
Strain Stability Analysis of the Stretchable UV Blind Photodetector Based on β-Ga_2_O_3_ Nanowires. (**a**) Schematic diagram of necking in the device under tensile strain. (**b**) Changes in electrode spacing and light-sensitive area with tensile strain, obtained through COMSOL simulation. (**c**) Variations in photoelectric current and dark current of the device under different tensile strains. (**d**) Changes in 𝑅 and 𝑃DC𝑅 of the device under different tensile strains. (**e**) Single-cycle I-T curves of the device under different tensile strains. (**f**) Changes in photoelectric current and dark current of the device after 500 cycles of 0–50%−0% tensile strain. (**g**) Changes in photoelectric current and dark current rates of the device after 500 cycles of 0–50%−0% tensile strain.

To investigate the strain stability of stretchable solar-blind UV photodetectors based on β-Ga₂O₃ nanowires, it is necessary to test their photoelectric performance parameters under applied tensile strain. The design ensures that the wrinkles of the Ag nanowire electrodes are parallel to the direction of tensile strain to minimize damage to the electrodes, as shown in the upper part of Fig. 4(a). To explore the impact of tensile strain on the device’s responsivity and other specific photoelectric performance parameters, it is essential to first clarify the change in the device’s light-sensitive area during stretching. As tensile strain increases, the rate of change in the device’s light-sensitive area can be approximated by the rate of area change of the PDMS substrate. There is a notable necking effect in elastomer under tensile strain. By simulating the deformation of PDMS under tensile strain with COMSOL (as shown in the lower part of Fig. 4(a)), the width change due to necking in the elastomer can be determined, which allows the calculation of the change rate in the device’s light-sensitive area. The width and area change rates of PDMS with increasing tensile strain are shown in Fig. 4(b). It can be seen that the width change rate due to necking in the elastomer reaches a maximum of 29.3%, and due to necking, the area only increases by 41.4% when stretched by 100%. The change in area S also affects the device’s *PDCR* and *R*, and their variations with increasing tensile strain are shown in Fig. 4(c). Both *PDCR* and *R* experience different degrees of decline, which is a combined effect of strain on the device’s light and dark currents and changes in the device’s light-sensitive area. The trend in *R* exhibits three distinct stages: a stable phase where photoelectric performance is almost unaffected for strains up to 10%, a failure phase with a significant drop in responsivity for strains over 60%. At 50% strain, the device’s *R* is 9.31 mA/W, and *PDCR* = 123.1, which is a decrease of 21.1% and 16.7%, respectively, compared to the initial state.

Figure 4(d) shows that the dark current of the device continuously increases with increasing tensile strain, rising from 5.22 nA to 8.43 nA. At 100% tensile strain, the dark current increases by 64.6% compared to the 0% strain condition, but the dark current remains in the nanoampere range, which has a relatively minor impact on the device’s photoelectric performance.

As tensile strain increases, the change in the device’s photocurrent can be divided into three stages:

**Initial Stable Stage** (Strain up to 10%): During this stage, the peak photocurrent remains almost unchanged with increasing tensile strain. This is because a strain of up to 10% is insufficient to significantly affect the contact interface between the device electrodes and β-Ga₂O₃ nanowires.

**Maintained Stage** (Strain between 20 and 60%): In this stage, the peak photocurrent is noticeably lower than in the first stage, with a change rate of less than 20% compared to the initial value. However, the photocurrent change is relatively small and even shows an increasing trend. This is due to the increase in light-sensitive area caused by stretching, and moderate stretching does not significantly damage the nanowire structure or the contact interface between the electrodes and the nanowires. At 50% strain, the photocurrent reaches a maximum value of 0.735 µA for this stage, changing by only 3.4% compared to the initial value.

**Failure Stage** (Strain between 60 and 100%): In this stage, the device’s photocurrent drops abruptly by over 35.8%, leading to a significant reduction in photoelectric performance. This dramatic decrease is due to excessive tensile strain causing sudden failure in the contact between the Ag nanowire electrodes and β-Ga₂O₃ nanowires, and possibly causing the nanowires to break or the pathways formed by their interconnections to fail.

It is observed that performance significantly deteriorates at strains above 60%, leading to device failure. Therefore, to test the performance changes after cyclic tensile strain, the cyclic strain range is set from 0 to 50% to prevent irreversible failure due to excessive strain. The observed decrease in photocurrent and increase in dark current in our stretchable solar-blind UV photodetector after tensile strain cycling arise from microstructural changes in β-Ga₂O₃ nanowires, contact degradation at the electrode-nanowire interface, and substrate relaxation effects. Tensile strain induces partial cracking, alignment shifts, or dislocations in the nanowires, disrupting conductive pathways and reducing photoresponse. Simultaneously, repeated stretching weakens the Ag nanowire-β-Ga₂O₃ interface, increasing contact resistance and leakage currents under dark conditions. Additionally, the elastomeric PDMS substrate exhibits permanent set over cycles, slightly reducing the light-sensitive area by misaligning the nanowire network. Despite these factors, our device demonstrates robust performance with minimal degradation after extensive strain cycling.

For the β-Ga₂O₃ nanowire stretchable blind UV photodetector under a single-cycle tensile strain of 0–50%−0%, the I-T curves are shown in Fig. 4(e). At different tensile strains of 0–50%, the device’s response current still exhibits a trend of initially decreasing and then slowly increasing. This is a result of the combined effects of nanowire failure and increased light-sensitive area due to strain. During the 50%−0% strain release process, the response photocurrent peak shows an overall decreasing trend, with a slight increase in response current when returning to 0% strain. This is because some nanowires experienced irreversible failure, such as complete breaking, during the application of strain, while others experienced only reversible failure due to loss of contact. Irreversibly failed nanowires remain in a failed state even after strain release, and the reduction in light-sensitive area also contributes to the decrease in response current. After releasing the strain back to the 0% initial state, the reversibly failed nanowires recover light sensitivity and carrier pathways, leading to a slight recovery in response current. During the 0–50%−0% tensile strain single-cycle process, the device’s photocurrent change rate is within 12.2%. At 50% strain, the device’s photocurrent decreases by only 3.6% compared to the initial state. After a single cycle, the photocurrent with fully released strain is reduced by only 1.4% compared to the initial state. Therefore, the device’s performance remains relatively stable within a 50% tensile strain single-cycle process and after cycling.

After performing multiple cycles of tensile strain (0, 10, 50, 100, and 500 cycles), the photocurrent and dark current were tested after strain release. The relationship between the light-dark current values and the number of cycles is shown in Fig. 4(f) and (g). After 10 tensile strain cycles, the photocurrent decreased slightly from 0.76 µA to 0.73 µA, a reduction of 3.4%, while the dark current increased significantly from 5.1 nA to 5.9 nA, an increase of 15.8%. Following 50, 100, and 500 tensile strain cycles, although the photocurrent and dark current showed a more considerable change compared to the initial state without strain, with the photocurrent decreasing by about 5% and the dark current increasing by approximately 20%, the changes in photocurrent and dark current compared to the 10-cycle results were relatively small. The photocurrent change rate ranged from − 1 to 2.3%, and the dark current increased by a maximum of 6.7%. This indicates that after 10 cycles, the device’s photocurrent and dark current stabilized, and after 500 cycles, they remained nearly unchanged.

As there are no reports on stretchable solar-blind UV photodetectors currently available, a comparison of the strain cycle stability with other stretchable photodetectors for different wavelength ranges is presented in Table 3.

**Table 3:** Comparison of strain cycle stability of stretchable photodetectors: this work vs. Reported Oxide-Based photodetectors.

Device	Response	Strain	Cycles	$\:{\varvec{I}}_{\varvec{p}\varvec{h}\varvec{o}\varvec{t}\varvec{o}}$ change	Ref.
β-Ga_2_O_3_ NWs	254 nm	50%	500	3.9%	This work
ZnONWs	365 nm	80%	50	~ 7%	^35^
ZnO-PEDOT	365 nm	30%	1500	~ 10%	^36^

In summary, the stretchable blind ultraviolet photodetector based on β-Ga_2_O_3_ nanowires achieves a 𝑅 of 11.8 mA/W and a *PDCR* of 147.2 under 1600 µW/cm² light power density and 20 V bias, with no applied tensile strain. The change in the photodetector’s performance under 0–100% tensile strain can be divided into three stages: (1) a stable stage for strains less than 10%, (2) a maintenance stage for strains between 20% and 50%, and (3) a failure stage for strains greater than 60%. Under 50% tensile strain, after 0 to 10 cycles of strain, the photodetector’s performance slightly decreases, with the photogenerated current decreasing by 3.4% compared to the initial state. After 50 to 500 cycles, the photogenerated current changes by only − 1–2.3% compared to after 10 cycles, indicating that the device’s performance remains relatively stable with increased cycling. This stability is related to irreversible changes such as the complete fracture of nanowires and electrode contact interfaces during the initial few strains.

## Demonstration of the array application of stretchable solar-blind UV photodetectors with β-Ga₂O₃ nanowires

**Fig. 5:**
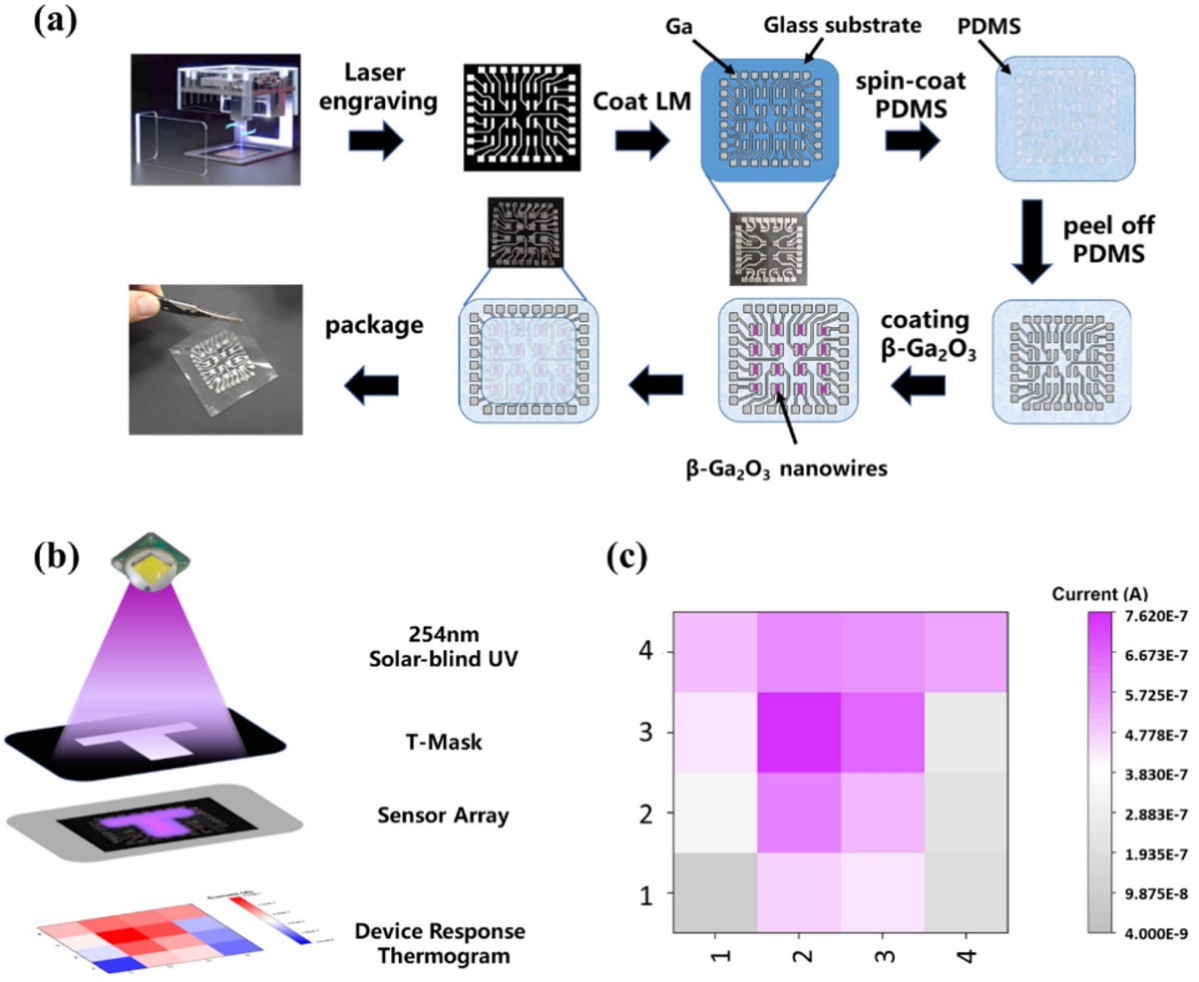
4 × 4 Stretchable solar-Blind UV Photodetector Array. (**a**) Fabrication Process Flowchart; (**b**) Testing Schematic Diagram; (**c**) Response Heatmap.

To verify the selective response of β-Ga_2_O_3_ nanowire-based stretchable blind ultraviolet detectors to the blind UV wavelength range, and to demonstrate the application of these devices in blind UV imaging, a 4 × 4 array of blind UV detectors was fabricated. The array was used to image patterned blind UV light. The overall array size is 40 mm × 40 mm, with each device, or pixel, measuring 4 mm × 3 mm, and the devices are spaced 4 mm apart. The fabrication process is illustrated in Fig. 5(a).

First, a laser engraver was used to etch the electrode and wire mask pattern on a 20 μm thick PET film, as shown in the figure. To prevent the device from being obstructed during testing with the B1500 A probe, the design includes extending the device electrodes to the edges of the circuit. Next, the PET mask was adhered to a smooth glass substrate, and a paste of liquid metal gallium was evenly applied to the gaps in the mask. A layer of PDMS was then spin-coated over this, and after curing, the electrode array was transferred to the PDMS substrate. Finally, β-Ga_2_O_3_ nanowires were placed between the two electrodes, and a thin layer of PDMS was spin-coated for encapsulation. This encapsulation layer enhances the robustness of the device but slightly reduces its response. However, due to the high transmittance of PDMS for the blind ultraviolet wavelength range, the performance of the device in detecting blind ultraviolet light is only minimally affected by the PDMS encapsulation. The final result is a 4 × 4 array of stretchable blind ultraviolet detectors based on β-Ga_2_O_3_ nanowires.

A 254 nm blind ultraviolet light source with a light power density of 1600 µW/cm² was applied to the device through a T-shaped optical mask while a bias voltage of 20 V was applied. The photoresponse current of each device in the array was tested, and the resulting response heat map of the array is shown in Fig. 5(b) and (c). The response heat map roughly outlines a T-shape, with a significant difference in response currents between the areas covered by the mask and those not covered. The highest response current reached 0.76 µA, while the lowest was only 0.09 µA. The variation in response current causes the heat map to resemble the T-shape of the mask, confirming the selective response of the device to blind ultraviolet light. Additionally, individual devices show good stability in detecting blind ultraviolet light under large strains. Therefore, this stretchable blind ultraviolet detector has promising applications in flame detection for rescue robots’ electronic skin and smart fire-fighting suits.

## Conclusion

To address the critical demand for flexible UV detection in firefighting robotics and safety systems, we have developed an innovative fabrication strategy combining electrospinning and phase transition to mass-produce high-performance β-Ga₂O₃ nanowires. The resulting stretchable solar-blind UV photodetector achieves remarkable optoelectronic characteristics, including an ultra-low dark current (5.12 nA), high photoresponse (0.76 µA), fast response/recovery times (0.8 s/0.36 s), and outstanding sensitivity (PCDR = 147.2, *R* = 22.5 mA/W). More significantly, the device demonstrates unprecedented mechanical robustness, maintaining 96.6% of its initial photocurrent under 50% tensile strain and showing merely 3.9% degradation after 500 strain cycles - representing the best strain stability reported to date for solar-blind UV detectors. We further validate the practical applicability of this technology through successful UV imaging using a prototype detector array, paving the way for next-generation conformal photodetectors in intelligent firefighting systems and other extreme-environment applications.

## Electronic supplementary material

Below is the link to the electronic supplementary material.

Supplementary Material 1

## Data Availability

The data in the article is available, please contact Jie SHANG(shangjie@nimte.ac.cn) and Ziyin XIANG(ziyinx92@163.com).
