# Supplementary material for: Self-supported β-Ga2O3 nanowires and for stretchable solar-blind UV photodetectors
Source: Sci Rep. 2025 May 20;15:17416. doi: 10.1038/s41598-025-02563-1 (PMC12092803; doi:10.1038/s41598-025-02563-1)
Supplement: Supplementary file 1 — Supplementary Material 1 [file 41598_2025_2563_MOESM1_ESM.docx]

**Supplementary Material**

Self-supported β-Ga_2_O_3_ nanowires and for stretchable solar-blind UV photodetectors

Wuxu Zhang^a^, Ziyin Xiang^a,b*^, Tengfei Ma^a^, Baoru Bian^a^, Jinyun Liu^a^, Yuanzhao Wu^a^, Yiwei Liu^a^, Jie Shang^a*^, Run-Wei Li^a,c*^

^a^ Ningbo Institute of Materials Technology and Engineering, Chinese Academy of Sciences, Ningbo 315201, China

^b^ College of Information and Intelligence Engineering, Zhejiang Wanli University, Ningbo, 315100, China.

^c^ Eastern Institute of Technology, Ningbo 315200, China

Corresponding author: Ziyin Xiang (ziyinx92@163.com); Jie Shang (shangjie@nimte.ac.cn); Run-Wei Li (rwli@eitech.edu.cn)


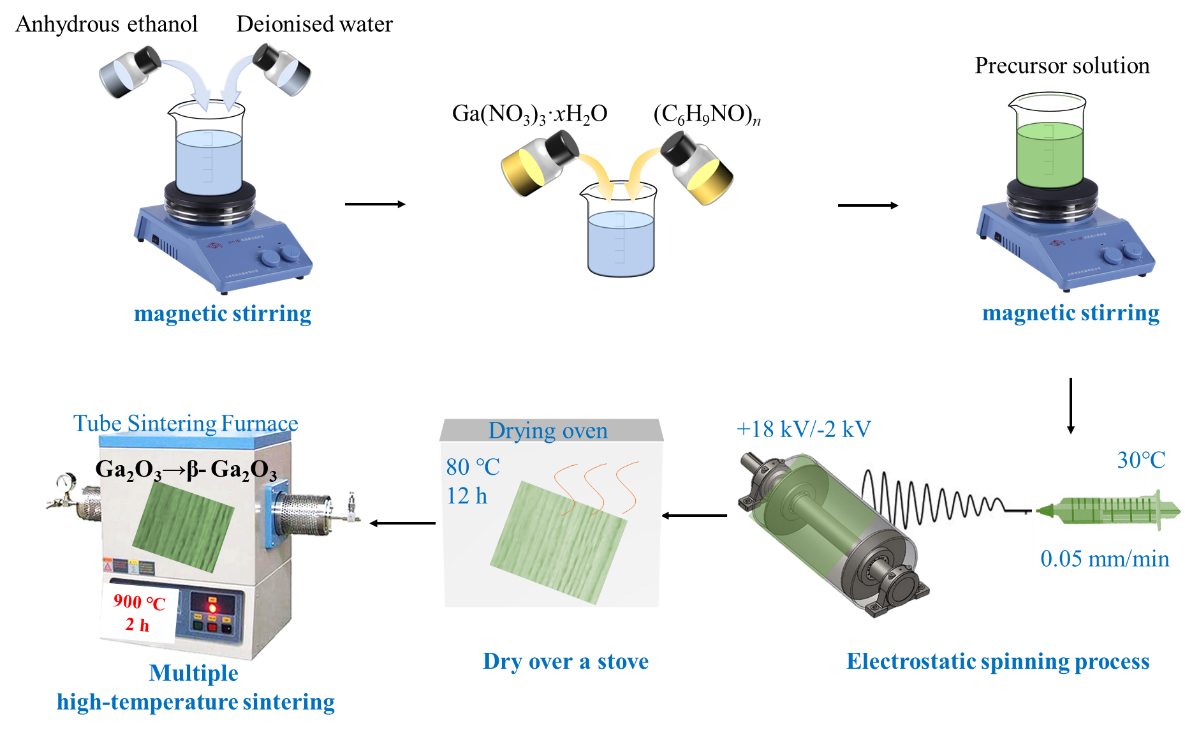


Figure S1. β-Ga2O3 nanowire preparation steps.


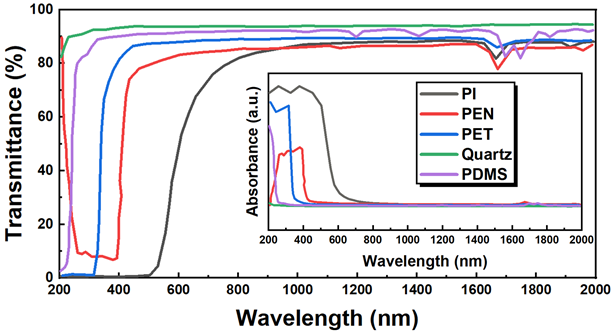


Figure S2. Transmission and Absorption Spectra of Individual Elastomers

The optoelectronic performance of the blind-UV detectors in this study was primarily tested using a self-built optoelectronic testing system. The system mainly includes a B1500A semiconductor parameter analyzer, a 254 nm blind-UV light source, a UV power meter, a UVC probe, and a timer, as shown in Figure S3.

The B1500A semiconductor parameter analyzer from Keysight was used to precisely measure the current-voltage characteristics of the devices. This instrument is commonly used for characterizing the electrical performance of detectors. A 254 nm and 365 nm light source from Zhongshan Zigu Lighting was employed, with a non-linear variation in light power density from 13 to 0.1 mW/cm² over an irradiation distance of 0-90 mm, and a spot diameter range of 18-85 mm. An LS125 UV power meter from Linshang Technology, paired with a UVCLED-X0 probe, was used to measure the UV light power density at specific distances to obtain the required testing light power density. The UV power meter displays power values, energy values, and maximum and minimum power values in real-time. The probe's spectral response range of 230-280 nm covers the blind-UV range, with a measurement range of 0-200 mW/cm² and an aperture diameter of 10 mm, meeting the diameter requirements for UV light power density measurement. A cycle timer controller from Xunyi Electronics was used to control the periodic switching of the UV lamp, with time control precision in the millisecond range.


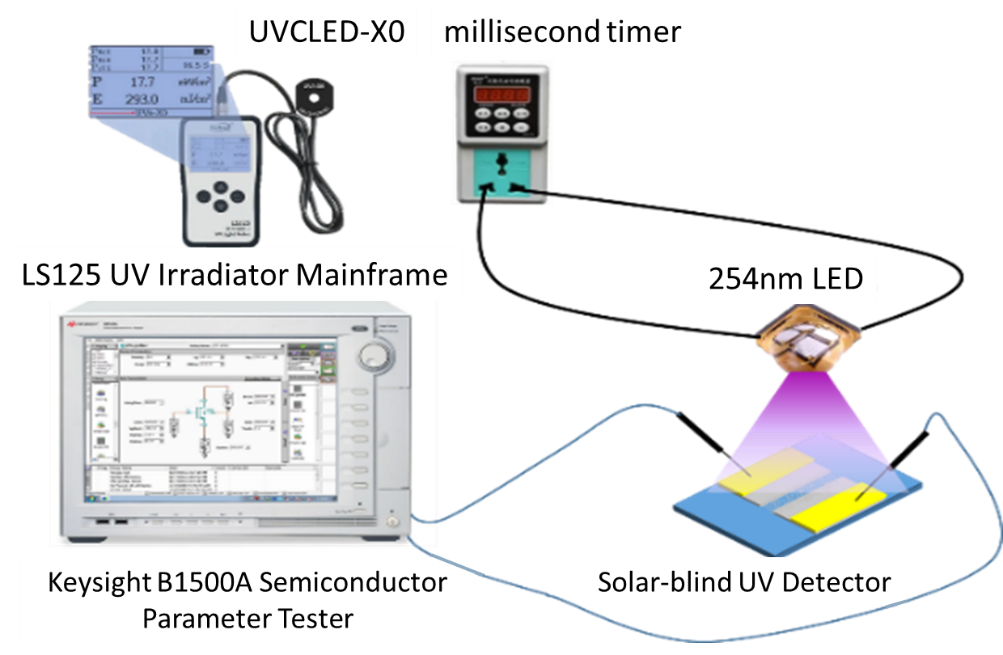


Figure S3. Self-built optoelectronic test system

To investigate the changes in optoelectronic performance of blind-UV photodetectors under bending and tensile strain, a simple and compact strain application and cycling system is required. The strain application system used in this study is based on a precision linear T-type slide module with a miniature handwheel. The strain cycling system consists of a miniature electric precision linear T-type slide, a power supply, a driver, and a programmable controller, as shown in Figure S4.

In this study, the precision handwheel T-type small slide from Haijia Jiachuang was used for strain application. This slide has a precision of 0.1 mm and a travel range of 0-200 mm, and is manually adjustable for stable operation. The strain cycling system consists of a stepper motor-driven T-type screw electric slide from Haijia Jiachuang, an S-120-24 power supply, a DM420S stepper driver, and a CL-01A single-axis programmable controller, which allows for the setting of speed, distance, and number of cycles.

For the specific experiments, the sample dimensions are 10 mm in width, 50 mm in length, and less than 1 mm in thickness. The sample is fixed onto the handwheel slide for the application of 0%-100% tensile strain, with a step size of 10% and a slide travel range of 50-100 mm. For strain cycling tests, samples of the same dimensions are fixed onto the slide controlled by the stepper motor. The programmable controller is used to set the slide speed to 10 mm/min, the travel distance to 50-75 mm, and the number of cycles to 10, 50, 100, and 500. After cycling, the optoelectronic performance of the device is tested using the optoelectronic testing system in the no-strain condition.


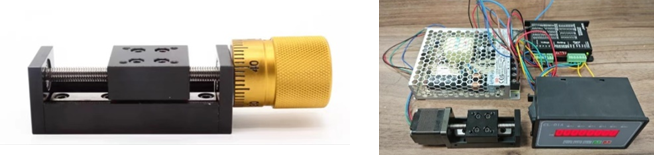


Figure S4. Strain Application and Cycling System

After calcination, the surface of the nanowires shows densely distributed protrusions. Selected area electron diffraction (SAED) and energy-dispersive X-ray spectroscopy (EDS) tests on individual protrusions of a single nanowire, as shown in Figure S5(a), reveal the presence of carbon (C) in addition to gallium (Ga) and oxygen (O). The presence of carbon can be attributed to the high-temperature decomposition residues of polyvinylpyrrolidone (PVP), which contains carbon (C), hydrogen (H), nitrogen (N), and oxygen (O).

The material underwent multiple calcination steps. After each calcination, high-purity oxygen was continuously introduced once the temperature reached above 500°C, and the temperature was maintained at 900°C with high-purity oxygen for 2 hours. High-temperature oxidation in an oxygen-rich environment was used to remove carbon residues from the high-temperature decomposition of PVP. After high-temperature oxidation, the nanowires exhibited scattered pits, and carbon was no longer detected in the selected area EDS electron diffraction, as shown in Figure S5(b). Compared to before multiple calcinations, it can be concluded that high-temperature oxidation effectively removed the carbon residues from the nanowires.


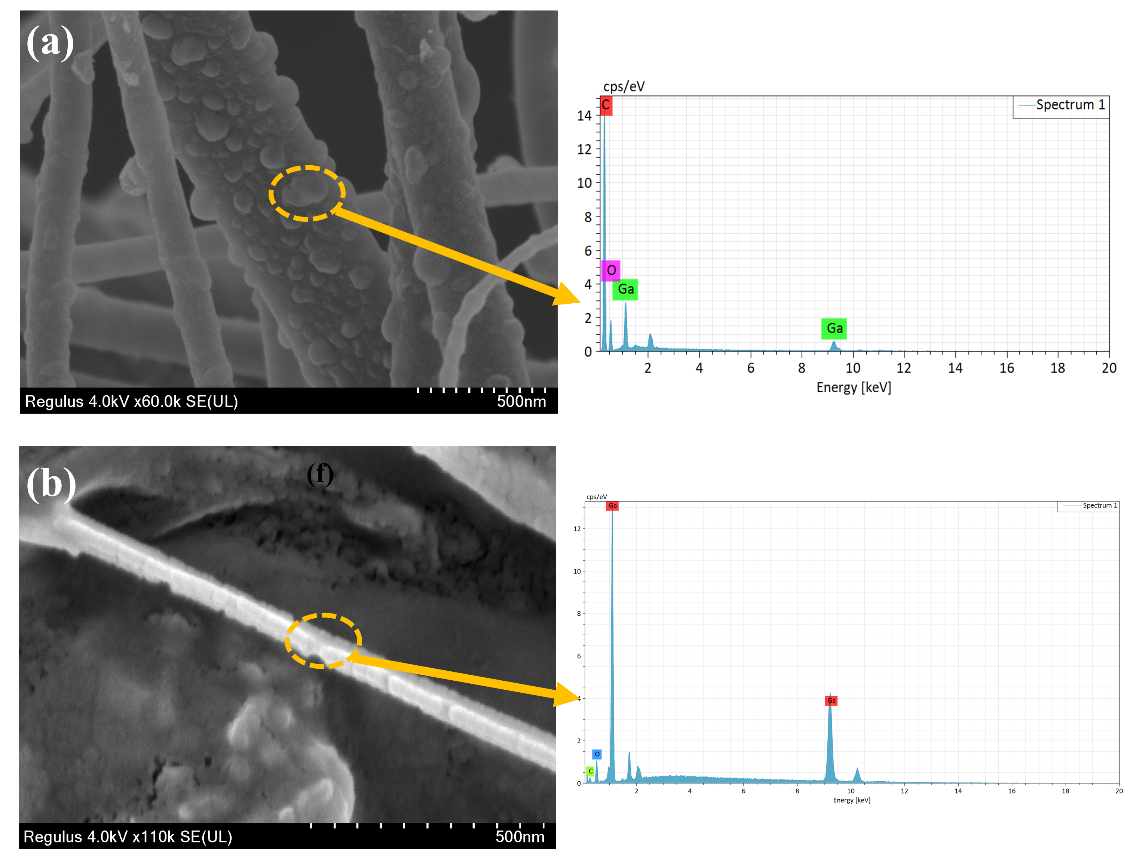


Figure S5. (a) Microscopic morphology of the nanowires and selected area EDS characterization of defects in the nanowires after a single calcination; (b) Microscopic morphology of the nanowires and selected area EDS characterization of defects in the nanowires after multiple high-temperature oxidation calcinations.


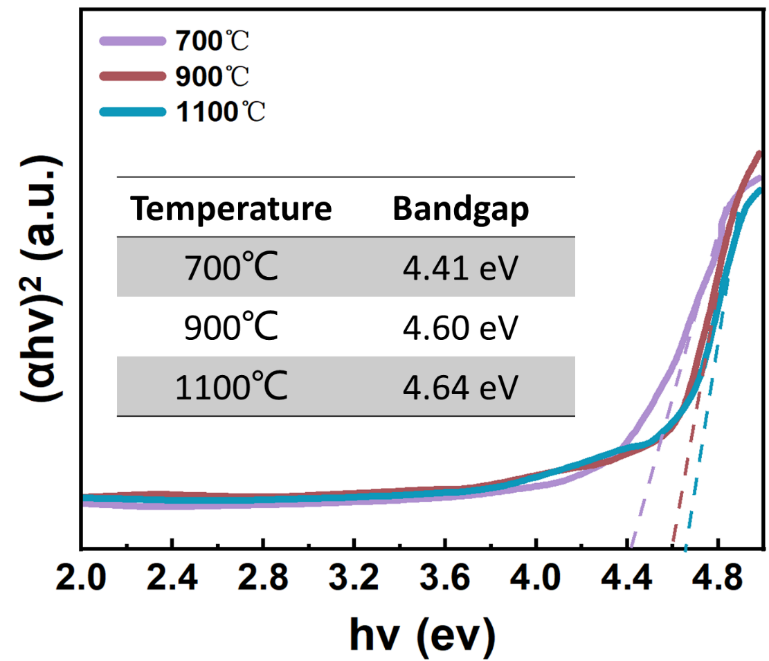


Figure S6. Relationship between *(αhv)^2^* and *hv* for nanowire materials prepared at different calcination temperatures.
